# Urban malaria and its determinants in Eastern Ethiopia: the role of *Anopheles stephensi* and urbanization

**DOI:** 10.1186/s12936-024-05126-3

**Published:** 2024-10-09

**Authors:** Hailu Merga, Teshome Degefa, Zewdie Birhanu, Ephrem Abiy, Ming-Chieh Lee, Guiyun Yan, Delenasaw Yewhalaw

**Affiliations:** 1 Department of Epidemiology, Institute of Health, Jimma University, Jimma, Ethiopia; 2 School of Medical Laboratory Sciences, Institute of Health, Jimma University, Jimma, Ethiopia; 3 Tropical and Infectious Diseases Research Center (TIDRC), Jimma University, Jimma, Ethiopia; 4 Departement of Health, Behavior, and Society, Faculty of Public Health, Jimma University, Jimma, Ethiopia; 5 Abt Global PMI Evolve Project, Addis Ababa, Ethiopia; 6 Program in Public Health, University of California at Irvine, Irvine, USA

**Keywords:** Urban malaria, Urbanization, Matched-case control, *Anopheles stephensi*, Eastern Ethiopia

## Abstract

**Background:**

Malaria prevention and control strategies have been hampered by urbanization and the spread of *Anopheles stephensi*. The spread of this vector into Africa further complicates the already complex malaria situation, that could put about 126 million Africans at risk of infection. Hence, this study aimed to assess the determinants of urban malaria, focusing on the role of urbanization and the distribution of *An. stephensi* in Eastern Ethiopia.

**Methods:**

A matched case control study was conducted among febrile urban residents of Dire Dawa (malaria positive as cases and negative as a control). A capillary blood sample was collected for parasite identification using microscopic examination and an interviewer administered questionnaire was used to collect additional data. Centers for Disease Control and Prevention miniature light traps (CDC-LT) and Prokopack aspirator were used to collect adult mosquito vectors from the selected cases and control houses to identify the mosquito vector species. Then, the data were exported to STATA for analysis. Conditional logistic regression was done to identify determinants, and principal component Analysis (PCA) was done for some independent variables.

**Results:**

This study enrolled 132 cases and 264 controls from urban setting only. Of the 132 cases, 90 cases were positive for *Plasmodium falciparum*, 34 were positive for *Plasmodium vivax* and 8 had mixed infections. All cases and controls were similar with regard to their respective age and sex. Travel history (AOR: 13.1, 95% CI 2.8–61.4), presence of eves and holes on walls (AOR: 2.84, 95% CI 1.5–5.5), history of malaria diagnosis (AOR: 2.4, 95% CI 1.1–5.3), owning any livestock (AOR: 7.5, 95% CI 2.4–22.8), presence of stagnant water in the area (AOR: 3.2, 95% CI 1.7–6.1), sleeping under bed net the previous night (AOR: 0.21, 95% CI 0.1–0.6) and knowledge on malaria and its prevention (AOR: 2.2, 95% CI 1.2–4.1) were determinants of urban malaria infection. About 34 adult *Anopheles* mosquitoes were collected and identified from those selected cases and control houses and 27 of them were identified as *An. stephensi*.

**Conclusion:**

Among the cases, the dominant species were *P. falciparum*. This study identified travel history, house condition, past infection, livestock ownership, stagnant water, bed net use, and malaria knowledge as determinants of infection. This study also found the dominance of the presence of *An. stephensi* among the collected mosquito vectors. This suggests that the spread of *An. stephensi* may be impacting malaria infection in the study area. Hence, strengthening urban-targeted malaria interventions should be enhanced to prevent and control further urban malaria infection and spread.

## Background

Malaria remains a major public health problem in the tropical countries of the world, especially in sub-Saharan Africa. *Plasmodium falciparum* and *Plasmodium vivax* are the most prevalent, and *P. falciparum* is the most virulent species [1–4]. The co-existence of these two species makes malaria control more complicated [5–7]. According to the World Malaria Report 2023, there were an estimated 249 million malaria cases globally in 85 malaria endemic countries, an increase of 5 million cases compared with 2021. Most of these cases were coming from countries in the World Health Organization (WHO) African Region [8].

The WHO and UN (United Nations) Habitat indicated that by 2050 nearly 7 out of 10 people globally will live in cities and urban settings. They developed the global framework for the response to malaria in urban areas, which indicated that although many will benefit from their urban status, rapid and unplanned urbanization can have negative health, social, and environmental impacts [9].

Unplanned growth of urbanization and the spread of the invasive malaria vector *Anopheles stephensi* make malaria control in urban areas difficult. The spread of this vector into Africa further complicates the already complex malaria situation. Modeling studies indicated that it could put about 126 million additional Africans at risk of contracting malaria and increase the malaria cases in Ethiopia by 50% [10, 11]. The characteristics of this vector make its control challenging; it quickly adapts to the local environment, surviving extremely high temperatures during the dry season, when malaria transmission usually reaches a seasonal low. This vector appears to swiftly acclimate to its surroundings and thrives in man-made water containers in urban settings. Moreover, the vector has also exhibited resistance to several classes of insecticides. If uncontrolled, its spread across the Horn of Africa, combined with rapid and poorly planned urbanization, may increase the risk of malaria outbreak in African cities [8, 10, 12–17].

Urbanization has altered health patterns in certain areas, making infectious disease risks in urban environments different from those in rural ones [18]. Despite their growing urbanization, many African countries, including Ethiopia, are still plagued by poor housing, inadequate sanitation, and poor surface water drainage, all of which would be favorable environments for mosquito proliferation. Since it is generally believed that economic development in urban areas leads to better living conditions, such as improved housing, drainage systems, and environmental changes, making urban areas not conducive for breeding of malaria vectors, malaria has been overlooked in urban settings for centuries [19]. Even though some old studies showed that urbanization decreases the morbidity and mortality from malaria [20–23], other recent study showed urbanization as a risk of malaria transmission, especially after the spread of urban malaria vector species [18]. Urbanization has significant epidemiological, entomological, parasitological, and behavioural implications on malaria risks. The global patterns of disease and mortality, including malaria, will change due to a shift in the human population from rural to urban areas. Urban malaria is projected to worsen as unplanned urbanization proceeds which increases the number of habitats where malaria vectors might breed, raising the danger of exposure to mosquito bites and malaria transmission [14, 20, 24–27].

There have been numerous attempts to assess the burden of malaria in Africa in general and in Ethiopia in particular, but none has taken into account how the expansion of unplanned urbanization may alter disease transmission and outcomes, and consequently death and morbidity estimates. Besides, the epidemiology of urban malaria since the spread of a new mosquito vector species, *An. stephensi*, in sub-Saharan Africa, including Ethiopia, is not well studied [12]. Additionally, there is no information on whether cases diagnosed in urban areas in Ethiopia lately came from nearby rural areas or if they were the consequence of transmission within the urban area. For urban malaria control strategies to be successful, it must be directed at the urban areas where infections are occurring. On the other hand, if malaria cases are coming from rural regions, control strategies must target the vectors, breeding grounds, and affected people there. If infections are contracted in cities, similar actions must be targeted differently. Because of this, effective urban malaria control depends on accurate clinical and epidemiologic data [18]. Hence, identifying the effect of urbanization and new spread of mosquito vector species on the Epidemiology of malaria in urban settings is crucial in the intended global as well as national malaria elimination plan. Therefore, this study identified urban-associated socioenvironmental determinants of urban malaria in Eastern Ethiopia.

## Methods

### Study design and setting

**Fig. 1:**
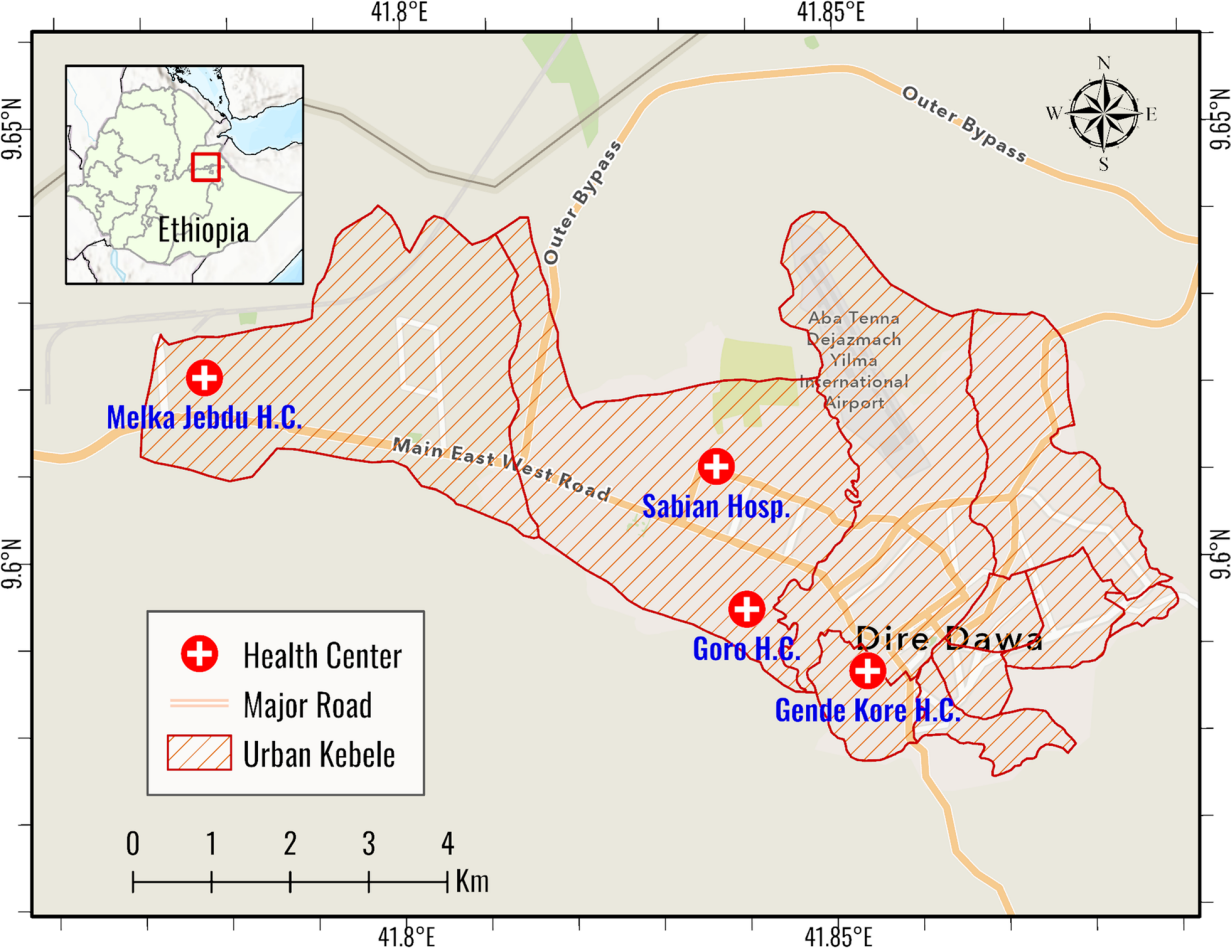
Map of the study area (Dire Dawa, 2023)

A matched case control study (1:2) was conducted between May 7 and May 30, 2023, in Dire Dawa city administration, located in the eastern part of Ethiopia, which is 515 km away from Addis Ababa, the capital of Ethiopia. Dire Dawa is one of the two city administrations in the Federal Democratic Republic of Ethiopia with about 465,000 populations. It is located between 90 28.1″ N and 90 49.1″ N latitude and between 410 38.1″ E and 420 19.1″ E longitude. There are two public hospitals (Dilchora Referral Hospital and Sabian Primary Hospital), six private hospitals (1 general and 5 primary hospitals), 10 health centers and 59 private clinics in the city (Fig. 1).

### Population

All febrile patients who visited the health facilities were the source population. For this matched case control study, cases were urban resident febrile patients positive for any malaria species and controls were urban resident febrile patients free of any malaria infection after diagnosis with microscopy. Febrile patients who were unable to respond or had communication problems, or unwilling to participate were excluded from this study. Febrile patients who visited the health facilities, either tested positive or negative, from rural areas were excluded from the case control study. Cases were defined as clients from urban areas only who had malaria illness by meeting three criteria: either an axillary body temperature ≥ 37.5 °C or history of fever (≤ 48 h), a positive blood smear by microscopy for *Plasmodium* infection (any parasite density) and a positive PCR test for *Plasmodium* infection*.* Controls were defined as individuals from urban settings of the same age group and same sex who came to health facilities with or without fever and who were negative for *Plasmodium* infection. Cases were individually-matched to two controls by age and sex. To collect adult *Anopheles* mosquitoes from chosen cases and control households, Centers for Disease Control and Prevention small light traps (CDC-LT) and Prokopack aspirators were employed.

### Sample size and sampling technique

The sample size for this study was determined using Stata software by considering the following sample size calculation for matched case control study design [28, 29] and taking the variable share bed net at night from the previous study [30], considering the proportion of discordant pairs and concordant pairs (26% proportion of cases exposed and 13.8% proportion of controls exposed), 84% power and 95% confidence level. Accordingly, with the addition of 5% non-response rate, the total sample size determined for this study was 396 (132 cases and 264 controls). Once the number of cases was determined and selected, a comparable or matched control was selected from the same health facility among febrile patients with negative results. Assuming that all of the health facilities were likely to be homogeneous in receiving clients, proportional allocation was done based on the average of the previous six-month report of malaria cases. Data were collected from cases and controls in the four selected health facilities as well as from the cases and controls households for an average of one month.

### Measurements

#### Wealth index

Household wealth index was assessed using Stata software version 16 with the following wealth index parameters: household items, cooking place, own livestock and their type, number of rooms, floor and roof materials of the house, energy sources and sources of drinking water. These scores are derived using Principal Component Analysis (PCA). Variables that exhibited low variation were excluded from the analysis. Then, the rest of the variables were considered for the final analysis to divide the distribution into five equal categories [31–33].

#### Knowledge

To assess the participants’ knowledge on malaria, its control and prevention, 12 knowledge related questions were used. An overall knowledge score was calculated by adding all scores for each respondent for all knowledge related questions. Then, mean was computed for those 12 variables and study participants who scored equal to or above the mean value were considered to have good knowledge about malaria, whereas those with scores less than the mean value were considered to have poor knowledge about malaria control and prevention.

#### Attitude

Attitude was assessed using 13 attitude related Likert scale questions. An overall attitude score was calculated by using principal component analysis and finally dichotomized as poor and good.

### Data collection tool and procedure

Data were collected by ODK software with the tool developed from different relevant and related literatures. Variables used for household wealth index construction were developed on the basis of literatures and the Ethiopian Demographic and Health Survey [19–21, 25, 27, 33–43]. The questionnaire was translated to both Afan Oromo and Amharic languages and back translated to English to check for consistency. Data was collected by trained and experienced data collectors/laboratory technicians, who were assigned to the health facilities until the sample size for each health facility was attained.

### Blood sample collection and laboratory analysis

A capillary blood sample was collected from each study participant following standard operation procedures. Both thick and thin blood smears were prepared on a clean, labeled microscopic slide for each selected study participant. Then, the thin film was fixed with absolute methanol and blood smear slides were transported to the laboratory of Tropical and Infectious Diseases Research Center (TIDRC) of Jimma University. Finally, the slides were stained with 10% Giemsa for 10 min, air dried, and examined microscopically by an experienced laboratory technologist. The slides were considered negative if no parasite was seen after examining 100 high power fields.

To ensure the quality of data, pretest was done among 5% of the sample size at Laga Hare Health center in Dire Dawa city. Training for data collectors (interviewers, laboratory technologists and supervisors) was given on how to collect data using ODK software, data collection approach and how to handle the sample as well as how to conduct laboratory investigations using standard operating procedures. As part of quality control, all positive blood smear slides and 10% of the negative slides were re-examined by another blinded senior laboratory technologist.

### Data processing and analysis

The Strengthening the Reporting of Observational Studies in Epidemiology (STROBE) [44] checklist for observational studies was used to analyze and report data. Data were downloaded from the Open Data Kit (ODK) server and appropriate cleaning was done before further analyses using Stata version 16 (Stata Corp. College Station, TX, USA). Conditional logistic regression was conducted for the cases individually-matched to two controls by age and sex. Because the analysis was matched on age and sex, the association between age and the odds of malaria was not reported. Principal component Analysis, a statistical approach used to reduce large data sets into smaller data sets without losing much of the original sample data, was used to compute the wealth Index and Attitude Variables.

The normality of continuous variables was checked. Categorical variables were reported by frequency and percentages. Proportions of each exposure variable among cases and controls were calculated. Using conditional logistic regression, variables associated with the outcome variable with p-value < 0.25 in bivariable analysis were included in the multivariable analysis. The matched Adjusted Odds Ratio (AOR), and their corresponding 95% Confidence Interval (CI) and p-values < 0.05 were calculated to measure the significance of the association. Multicollinearity was checked with Variance Inflation factor (VIF) and it indicates the absence of collinearity (VIF = 1.28). The parsimonious model fitness was checked by Hosmer and Lemeshow goodness of fit test and a P-value of 0.67 was obtained.

## Results

### Sociodemographic characteristic of participants

A total of 396 study participants (132 cases and 254 controls) were enrolled in the study. All cases and controls were similar with their respective age and sex. The median age of the respondents was 25 years for both cases and controls. The minimum and maximum ages were 12 and 65 years, respectively. Oromo were the dominant ethnic group in both cases (56.8%) and control (45.5%). Regarding their educational status, nearly half of the cases (47.7%) and more than one third of controls (42.1%) attended primary school. Similarly, more than one fourth of the cases (31.1%) and controls (25.8%) were daily laborers/construction workers. After Principal Component Analysis, the study showed that 20.45% of the cases and 20.83% of the controls were in the lowest wealth quantile range (Table 1).

**Table 1:** Socio-demographic and socio-economic characteristics of respondents in Dire Dawa, Eastern Ethiopia, 2023 (N = 396)

Variable	Variable category	Cases, n (%) (N = 132)	Controls, n (%) (N = 264)
Age		Mean (SD) = 28.12 (11.5)	Mean (SD) = 28.3(11.01)
Sex	Male	97 (73.5)	194 (73.5)
	Female	35 (26.52)	70 (26.52)
Ethnicity	Oromo	75 (56.8)	120 (45.5)
	Amhara	30 (22.73)	97 (36.74)
	Somali	5 (3.79)	19 (7.2)
	Gurage	8(6.1)	10 (3.8)
	Tigre	8(6.1)	11 (4.2)
	Others^a^	6(4.5)	7 (2.65)
Religion	Muslims	75 (56.82)	135 (51.33)
	Orthodox Christian	44 (33.3)	113 (42.97)
	Protestant	13 (9.8)	16 (6.1)
Marital status	Single	74 (56.1)	132 (50.6)
	Married	51 (38.6)	118 (45.2)
	Divorced	3 (2.27)	8 (3.01)
	Widowed	4 (3.03)	6 (2.3)
Educational status	Never attend	19 (14.4)	31 (11.7)
	Primary (1–8)	63 (47.7)	111 (42.1)
	Secondary (9–12)	37 (28.03)	101 (38.3)
	TVET	3 (2.3)	5 (1.9)
	Degree and above	10 (7.6)	16 (6.1)
Occupational status	Trader	16 (12.12)	40 (15.15)
	Employed (Government and private)	13 (9.85)	24 (9.1)
	Students	31 (23.5)	62 (23.5)
	Driver	7 (5.3)	20 (7.6)
	Daily laborers/construction workers	41(31.1)	68 (25.8)
	House wife and unemployed	16 (12.12)	42 (15.9)
	Others^b^	8 (6.1)	8 (3.03)
Family size	≤ 2	35 (26.5)	66 (25.0)
	3–5	75 (56.8)	164 (62.1)
	≥ 6	22 (16.7)	34 (12.9)
Presence of child in the house hold	Yes	62 (46.97)	86 (32.6)
	No	70 (53)	178 (67.4)
Head of the house holds	Yes	37 (28)	81 (30.7)
	No	95 (79.97)	183 (69.3)
Wealth Index	Lowest	27 (20.45)	55 (20.83)
	Second	24 (18.2)	53 (20.1)
	Middle	24 (18.2)	55 (20.8)
	Fourth	29 (21.97)	50 (18.9)
	Highest	28 (21.2)	51 (19.3)

^a^Gammo, Wolayita, Hadiya and Hadare

^b^Religious leader, retired, etc.

### Knowledge and attitude towards malaria and its prevention

The general knowledge of the study participants about malaria and its prevention was assessed in this study. Accordingly**,** more than one third (40.9%) of cases and more than half (57.6%) of controls had good knowledge. Similarly, Principal Component Analysis was used to compute the attitude of respondents toward malaria and its prevention as well as treatment. Accordingly, nearly half (47%) of the cases and more than half of the controls (51%) had good attitude towards malaria, its prevention and treatment (Table 2).

**Table 2:** Knowledge of malaria and its prevention among study participants in Dire Dawa (2023)

Variables	Variable category	Cases, n (%) (N = 132)	Controls, n (%) (N = 264)
Ever heard of malaria	Yes	130 (98.5)	264 (96.9)
	No	2 (1.5)	8 (3.1)
knew mosquito-breeding sites	Yes	103 (78.03)	190 (71.97)
	No	29 (21.97)	74 (28.03)
Thought recently malaria vector affects urban population than rural popular	Yes	46 (34.8)	90 (34.1)
	No	86 (65.2)	174 (65.9)
Knew malaria vectors can breed in any artificial water container	Yes	62 (46.97)	92 (34.9)
	No	70 (53.03)	172 (65.1)
knew malaria transmission	Yes	87 (65.9)	217 (82.2)
	No	45 (34.1)	47 (17.8)
Which group of population is more affected by malaria	Children	56 (42.42)	136 (51.52)
	Travelers/migrants	35 (26.5)	63 (23.9)
	Adults	12 (9.1)	17 (6.4)
	Pregnant mothers	22 (16.7)	29 (10.97)
	Older people	7 (5.3)	19 (7.2)
Thought malaria prevention and control is possible	Yes	111 (84.1)	239 (90.5)
	No	21 (15.9)	25 (9.5)
Knew drugs prescribed for malaria	Yes	4 (3.03)	11 (4.2)
	No	128 (96.97)	253 (95.83)
Bought malaria drug without physician prescription	Yes	7 (5.3)	4 (1.5)
	No	125 (94.7)	260 (98.5)
Water container found in your compound	Yes	41 (31.1)	62 (23.5)
	No	91 (68.9)	202 (76.5)
knowledge (over all)	Poor	78 (59.1)	112 (42.42)
	Good	54 (40.9)	152 (57.6)
Attitude (over all)	Poor	70 (53.03)	129 (48.9)
	Good	62 (46.97)	135 (51.14)

### History of travel, malaria diagnosis and environmental factors

More cases 24 (18.2%) than controls 19 (7.2%) had history of travel in the last 2 weeks. Similarly, within the past one month, all of the cases and few controls (only 4) reported that they had history of travel outside Dire Dawa town and slept overnight there. On the other hand, about one-fourth 35 (26.5%) of the cases and less than one tenth of the controls 22 (8.3%) had history of malaria diagnosis. History of family members malaria diagnosis in the past one month was also assessed, and more than one fifth of the cases 29 (21.97%) and 12 (4.6%) of the controls had history of malaria diagnosis. One-fourth 33 (25%) of the cases and more than one-fourth of the controls 76 (28.8%) had any kind of bed net. Fewer number of cases 8 (6.1%) and one fourth of the controls 56 (21.2%) slept under bed net last night. A smaller number of cases 4 (3%) and 22 (8.3%) controls reported that they were using mosquito repellent skin lotion (traditional plant) for the prevention of malaria infection. Environmental factors like the presence of stagnant water in the nearby environment was assessed in this study, and there was stagnant water in the majority of the case’s (60.6%) and one fourth of the control’s 76 (28.8%) home or residential areas (Table 3). With CDC light trap and Prokopack traps, adult mosquito vectors were collected from the selected cases and the selected control houses. About 33 adult *Anopheles* mosquitoes were collected and 14 were identified as fed and 1 was half gravid. Accordingly, 27 of them were identified as *An. stephensi* and the rest were *Anopheles gambiae *sensu lato.

**Table 3:** History of travel and malaria diagnosis among study participants in Dire Dawa, Eastern Ethiopia (2023)

Variables	Category	Cases, n (%)	Controls, n (%)
History of travel in the last 2 weeks	Yes	24 (18.2)	19 (7.2)
	No	108 (81.8)	245 (92.8)
History of malaria diagnosis	Yes	35 (26.5)	22 (8.3)
	No	97 (73.5)	242 (91.7)
within one month, travelled outside town and slept overnight	Yes	24 (18.2)	4 (1.5)
	No	108 (81.8)	260 (98.5)
History of family members malaria diagnosis in the past one month	Yes	29 (21.97)	12 (4.6)
	No	103 (78.03)	252 (95.4)
Owned any bed net	Yes	33 (25)	76 (28.8)
	No	99 (75)	188 (72.2)
Ever sold or given away a mosquito net	Yes	7 (5.3)	20 (7.6)
	No	125 (94.7)	244 (92.4)
Household member purchase the nets	Yes	4 (3.03)	22 (8.3)
	No	128 (96.97)	242 (91.7)
Did you sleep under bed net last night?	Yes	8 (6.1)	56 (21.2)
	No	124 (93.9)	208 (78.8)
Used mosquito repellent skin lotion (traditional plant)	Yes	4 (3)	22 (8.3)
	No	128 (96.97)	242 (91.7)
vegetation/agricultural land in your living compound?	Yes	89 (67.2)	169 (64)
	No	43 (32.6)	95 (36)
Stagnant water found around your home/area	Yes	80 (60.6)	76 (28.8)
	No	52 (39.4)	188 (71.2)
Owned any live stock	Yes	28 (21.21)	10 (3.8)
	No	104 (78.8)	254 (92.6)
Proximity to water body	≤ 1 km	123 (93.2)	254 (96.2)
	> 1 km	9 (6.8)	10 (3.8)

### Determinants of urban malaria: the conditional logit model

To assess the predictors of urban malaria, conditional logistic regression was done to identify predictors in this study. Bi-variable analysis was performed to identify candidate variables for the final model, multivariable model (Table 4). Accordingly, presence of eves and other holes on the wall, knowledge on malaria and its prevention, history of malaria diagnosis, history of travel outside the town and slept overnight, slept under bed net last night, owning any livestock and presence of stagnant water in the area were statistically significant variables in the final model (Table 5).

**Table 4:** Bivariate analysis for the determinants of urban malaria in Dire Dawa, Eastern Ethiopia (2023)

Variable	Variable category	Cases, n (%) (N = 137)	Controls, n (%) (N = 250)	COR (95% CI)	p value
Religion	Muslims	75 (56.82)	135 (51.33)	1.0	
	Orthodox	44 (33.3)	113 (42.97)	0.7 (0.43–1.1)	0.12*
	Protestant	13 (9.8)	15 (5.7)	1.4 (0.65–3.2)	0.37
Marital status	Single	74 (56.1)	132 (50.6)	1.0	
	Married	51 (38.6)	118 (45.2)	0.62 (0.33–1.1)	0.12*
	Divorced	3 (2.27)	8 (3.01)	0.57 (0.13–2.56)	0.47
	Widowed	4 (3.03)	6 (2.3)	1.1 (1.5, 8.2)	0.93
Educational status	Never attend	19 (14.4)	31 (11.7)	1.2 (0.57–2.5)	0.64
	Primary (1–8)	63 (47.7)	111 (42.1)	1.0	
	Secondary (9–12)	37 (28.03)	101 (38.3)	0.63 (0.38–1.03)	0.07*
	Technical and vocational school and above	13 (9.8)	21 (7.9)	1.04 (0.48, 2.27)	0.92
Presence of child in the house hold	Yes	62 (46.97)	86 (32.6)	1.9 (1.2–2.9)	0.005*
	No	70 (53)	178 (67.4)	1.0	
Presence of eves and other holes on the wall	Yes	85 (64.4)	122 (46.2)	2.3 (1.5–3.7)	< 0.0001*
	No	47 (35.6)	142 (53.8)	1.0	
knowledge	Good	54 (40.9)	152 (57.6)	1.0	
	Poor	78 (59.1)	112 (42.42)	2.1 (1.3–3.3)	0.001*
History of travel in the last 2 weeks	Yes	24 (18.2)	19 (7.2)	2.9 (1.5–5.6)	0.001*
	No	108 (81.2)	245 (92.8)	1.0	
History of malaria diagnosis	Yes	35 (26.5)	22 (8.3)	3.8 (2.1–6.7)	< 0.0001*
	No	97 (73.5)	242 (91.7)	1.0	
History of travel outside the town and sleep overnight there	Yes	26 (19.7)	4 (1.5)	16.6 (5.6–49.4)	< 0.0001*
	No	106 (80.3)	260 (98.5)	1.0	
History of family members diagnosed of malaria	Yes	29 (21.97)	12 (4.6)	5.9 (2.9–12.3)	< 0.0001*
	No	103 (78.03)	252 (95.4)	1.0	
Purchased the nets	Yes	4 (3.03)	22 (8.3)	0.34 (0.11–1.01)	0.052*
	No	128 (96.97)	242 (91.7)	1	
Slept under bed net last night	Yes	8 (6.1)	56 (21.2)	0.23 (0.11–0.51)	< 0.0001*
	No	124 (93.9)	208 (78.8)	1.0	
Used mosquito repellent skin lotion (traditional plant)	Yes	4 (3)	22 (8.3)	0.35 (0.12–1.03)	0.056
	No	128 (96.97)	242 (91.7)	1.0	
Household proximity to the water source	≤1 km	123	254	2.02 (0.74, 5.5)	0.17*
	> 1 km	9	10	1.0	
Presence of water container in the compound	Yes	41 (31.1)	62 (23.5)	0.48 (0.303–0.76)	0.002*
	No	91 (68.9)	202 (76.5)	1.0	
Own any live stock	Yes	28 (21.21)	10 (3.8)	7.9 (3.4–18.5)	< 0.0001*
	No	104 (78.8)	254 (92.6)	1.0	
Stagnant water in the area	Yes	80 (60.6)	76 (28.8)	4.3 (2.7–6.8)	< 0.0001*
	No	52 (39.4)	188 (71.2)	1.0	

*shows significant at p < 0.05

**Table 5:** Multivariate analysis for the determinants of urban malaria in Dire Dawa, Eastern Ethiopia, 2023

Variable	Variable category	Cases, n (%) (N = 137)	Controls, n (%) (N = 250)	COR (95%CI)	AOR (95%CI)	p value
Presence of eves and other holes on the wall	Yes	85 (64.4)	122 (46.2)	2.3 (1.5–3.7)	2.84 (1.5–5.5)	0.002*
	No	47 (35.6)	142 (53.8)	1.0	1.0	
knowledge	Good	54 (40.9)	152 (57.6)	1.0	1.0	
	Poor	78 (59.1)	112 (42.42)	2.1 (1.3 -3.3)	2.2 (1.2–4.1)	0.014*
History of malaria diagnosis	Yes	35 (26.5)	22 (8.3)	3.8 (2.1–6.7)	2.4 (1.1–5.3)	0.029*
	No	97 (73.5)	242 (91.7)	1.0	1.0	
History of travel outside the town and slept overnight	Yes	26 (19.7)	4 (1.5)	16.6 (5.6–49.4)	13.1 (2.8–61.4)	0.01*
	No	106 (80.3)	260 (98.5)	1.0	1.0	
Slept under bed net last night	Yes	8 (6.1)	56 (21.2)	0.23 (0.11–0.51)	0.21 (0.1–0.6)	0.003*
	No	124 (93.9)	208 (78.8)	1.0	1.0	
Own any live stock	Yes	28 (21.21)	10 (3.8)	7.9 (3.4–18.5)	7.5 (2.4–22.8)	< 0.001**
	No	104 (78.8)	254 (92.6)	1.0		
Presence of stagnant water in the area	Yes	80 (60.6)	76 (28.8)	4.3 (2.7–6.8)	3.2 (1.7, 6.1)	< 0.0001**
	No	52 (39.4)	188 (71.2)	1.0	1.0	

* shows significant at p  < 0.05; ** shows significant at p < 0.001

## Discussion

Urbanization increasingly affects the epidemiological characteristics of infectious diseases. It has marked entomological, parasitological and behavioural effects on malaria risks, which in turn has a significant impact on the public health system [20, 45].

Due to the widespread belief that improved living circumstances are a result of urban economic growth, malaria is more frequently seen as a problem of the rural poor, and the illness has been less considered in urban settings for long a time. While cities in many African nations are growing quickly, majority of them are unplanned and pose a risk for many vectors breeding. Hence, the optimal control of urban malaria depends on accurate epidemiologic and entomologic information about transmission [18]. Even though many studies conducted in Ethiopia on risk factors of malaria, no study has yet been conducted on the determinants of urban malaria especially after the spread of *An. stephensi* in the horn of Africa. Therefore, this study investigated the determinants of urban malaria in Eastern Ethiopia.

The findings from this matched case control study indicated that the presence of eves and other holes on the house wall of the respondents was identified as a predictor of urban malaria. Houses with eves and holes increased the urban malaria morbidity compared to houses with no eves and holes (AOR (95% CI) 2.84 (1.5–5.5). This is similar with the finding from Arsi, Ethiopia [46] even though the study participants were among all who visited health facilities.

History of malaria diagnosis was also found to increase the odds of malaria morbidity in this study (AOR (95% CI) 2.4 (1.1–5.3). This can be linked with the relapse nature of some malaria species. This is not in line with the finding from southwestern Ethiopia [43]. The discrepancy might be attributed to the study site, as data for this study was generated only from urban settings, whereas data for that study was generated from both urban and semi-urban health institutions.

The general knowledge of participants on malaria and its prevention was also investigated in this study. Individuals who had poor knowledge on malaria and its prevention were about 2.2 (AOR (95% CI) (1.2–4.1) more likely to be infected when compared with those with poor knowledge. This finding is supported by finding from Accra, Ghana [47]. This might be due to the fact that both studies conducted in urban and similar factors were considered.

In urban settings, which serve as centres for human migration and mobility, a better knowledge of imported malaria cases is crucial [18]. Accordingly, history of travel was assessed in this study and found to increase the odds of urban malaria morbidity compared with those who had no history of travel (AOR (95% CI 13.1 (2.8–61.4)). The review from the International Center of Excellence for Malaria Research (ICEMR) supports this finding [18]. Similarly, this finding is in line with the findings from Malawi [35], a matched case control study from Uganda [38], Brazil [48] and South West Ethiopia [43]. This might be due to the fact that travelers are non-immune, lack health information about the area and unable to use mosquito repellents. Moreover, the diversity of *Plasmodium* species in urban settings could be on the rise due to the intensification of travels between different cities or regions [14].

In this study, sleeping under bed net the previous night decreased the odds of urban malaria infection compared with those non-utilized (AOR (95% CI) 0.21 (0.1–0.6)). A finding from south west Ethiopia [49], Southern Ethiopia [46], Uganda [38], Batu town, Ethiopia [39], Northwest Ethiopia [30], southern Ethiopia [50] and Amazon basin [51] supports this finding. The presence of any live stock in the house increased the odds of malaria morbidity in this study (AOR (95%): 7.5 (2.4–22.8). The crude analysis from Ziway-Dugda, southern Ethiopia [50] and finding from Indonesia [52] are in line with this study. The entomological studies from Djibouti [15], Malawi [24] and a review done at African level [10] support this finding. However, this study is in contrast with studies from southern Ethiopia [53] and south western Ethiopia [54] which might be due to the fact that the difference in the study population and the study design utilized.

Among the environmental factors, the presence of stagnant water in the nearby house has increased the odds of malaria morbidity (AOR (95%): 3.2 (1.7–6.1). This is in line with many observational studies from Ethiopia [30, 39, 49, 55], Zimbabwe [56] and a systematic review and meta-analysis [57]. This may be because stagnant water is a suitable breeding ground for *Anopheles* mosquitoes. But this finding is in contrast with the matched case control study from southern Ethiopia [50] which might be due to the fact that malaria exposure factors like the presence of stagnant water were lower both among the cases and controls.

In this study, *An. stephensi* was the dominant malaria vector species collected from those selected cases and controls households. This indicates that the impact and spread of this malaria vector in the horn of Africa [15, 17].

This study has the following strengths: both cases and controls were selected from the same outpatient department and the same diagnostic algorithm was used for both groups which minimized selection bias. Similarly, principal component analysis was performed for attitude and wealth index variables. Though it has those strengths, it has the following limitations: First, Long-lasting insecticidal nets (LLIN) use was reported for some of the cases and controls rather than observed, since the visits of selected cases and controls were made only during the day. Second, urbanicity was not assessed since this study was facility-based and data from households with regard to household density, land cover, vegetation amount, and nighttime light brightness were not collected. Misquotes were collected from only selected households due to resource limitation and expectation that it will be addressed in another study.

## Conclusion

The findings of this study highlight the determinants of urban malaria. The study indicates having travel history a month before testing increases the odds of urban malaria infection. Presence of eves and other holes on the wall, history of malaria diagnosis, owning any livestock, presence of stagnant water in the area, sleeping under bed net last night and knowledge about malaria and its prevention were identified as determinants of urban malaria. Similarly, this study identified *An. stephensi* as the dominant malaria vector species. This finding suggests that the spread of this mosquito species into urban areas may be impacting malaria infection rates. Hence, strengthening urban-center malaria intervention should therefore play a significant role in preventing and controlling further urban malaria expansion/spread. Moreover, further research to link household data with urbanization variables using principal component analysis and the collection of mosquito species from each household is recommended.

## Data Availability

The datasets used and/or analyzed during the current study are available from the corresponding author on reasonable request.

## Abbreviations

AOR: Adjusted odds ratio
COR: Crude odds ratio
WHO: World Health Organization
